# Bioprinting Au Natural: The Biologics of Bioinks

**DOI:** 10.3390/biom11111593

**Published:** 2021-10-28

**Authors:** Kelsey Willson, Anthony Atala, James J. Yoo

**Affiliations:** Wake Forest Institute for Regenerative Medicine, Wake Forest School of Medicine, Winston Salem, NC 27157, USA; kwillson@wakehealth.edu (K.W.); aatala@wakehealth.edu (A.A.)

**Keywords:** bioprinting, bioink, 3D printing

## Abstract

The development of appropriate bioinks is a complex task, dependent on the mechanical and biochemical requirements of the final construct and the type of printer used for fabrication. The two most common tissue printers are micro-extrusion and digital light projection printers. Here we briefly discuss the required characteristics of a bioink for each of these printing processes. However, physical printing is only a short window in the lifespan of a printed construct—the system must support and facilitate cellular development after it is printed. To that end, we provide a broad overview of some of the biological molecules currently used as bioinks. Each molecule has advantages for specific tissues/cells, and potential disadvantages are discussed, along with examples of their current use in the field. Notably, it is stressed that active researchers are trending towards the use of composite bioinks. Utilizing the strengths from multiple materials is highlighted as a key component of bioink development.

## 1. Introduction

In the last 16 years, bioprinting has taken the world of regenerative medicine by storm. The number of publications featuring bioprinting has grown exponentially as laboratories have adopted this new fabrication technology for their research. Traditional techniques for creating cellularized constructs, including non-cellularized scaffolds seeded post-fabrication and the implantation of non-cellularized constructs which recruit cells from the host, have severe limitations. It is difficult to adequately and uniformly seed these devices and place individually unique cell populations within the constructs. Those implanted without cells are challenged by cell migration within the scaffolds [1,2,3,4]. An alternative to these conventional manufacturing techniques, 3D printing, has several key strengths, including allowing users full control over the shapes and compositions of their printed components, including precise placement of diverse cell populations throughout the entirety of the construct. [5]. In addition, bioprinting allows users to choose/tune the carrier material or bioink used during printing. This allows fine control over chemical and physical environments specific to the end-use of each system. While these particular attributes have proven enticing to researchers who wish to pattern constructs with different cells, mechanical attributes, or chemical profiles, the realities of bioprinting have proven to be nuanced and complex, resulting in a broad field of study without a simple “push this button to print an organ” option. This article aims to give a broad overview of the biological components available as bioinks today, highlighting some of their many uses in the field.

## 2. Types of Bioprinters

Before selecting a bioink can begin, researchers must first ascertain two things: the environment they wish to replicate using the bioprinter and which type of bioprinter they will be using. Since the development of the first reported bioprinter in 2003, bioprinter technology has become a field of exponential growth [6]. While the field started with a single modified inkjet printer, today, several types of 3D printing are available to scientists [7,8]. These include inkjet, micro extrusion, laser-induced forward transfer (LIFT), digital light projection (DLP), selective laser sintering (SLS), and stereolithography. Choosing the appropriate printer for specific research is one of the first major decisions to be made. Each printer type requires different attributes from their bioink regarding viscosity, material type, and bonding mechanisms. This article will focus on bioinks developed for micro-extrusion and DLP printing, two commonly used systems for developing macro-constructs with cellular components.

**Figure 1:**
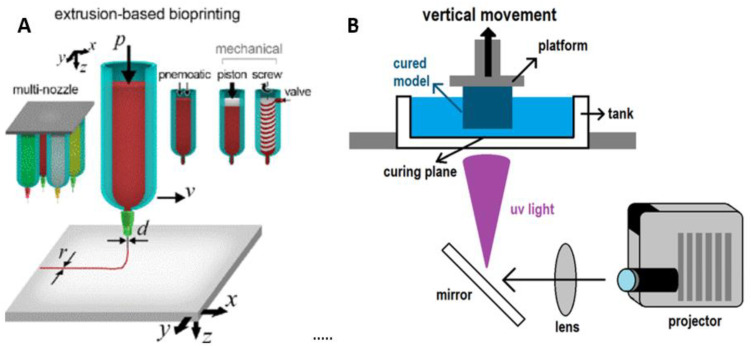
Diagrams showing the working mechanisms of micro-extrusion and DLP printers. (**A**) A micro-extrusion printer with examples of different extrusion nozzles and drivers (mechanical, pneumatic) along with the build platform [20]. (**B**) A DLP printer showing the orientations of the light source, curing plane, fabricated object, and build plate. Reprinted with the permission from Ref. [20]. Copyright 2018 AIP Publishing.

Micro-extrusion printers are one of the more common 3D printers used today, popular among both hobbyists and professionals. These printers consist of motor-driven axes, a media reservoir, and an extrusion orifice (Figure 1A). To be labeled as a micro-extrusion printer, the orifice should measure less than 1 mm in diameter. This includes nearly all extrusion printers used with cells as researchers move towards higher resolutions in the goals of recapitulating native cellular arrangements. The extrusion can be driven either through tunable pneumatic or mechanical pressure depending on the viscosity of the printing material [9,10,11]. Importantly, for researchers working with cells, cellular viability must be maintained. This means that extrusion bioinks should be composed of non-cytotoxic materials that can be tuned to minimize shear stress due to printing pressures, as high shear stress leads to decreased cellular viability [12,13,14]. Another critical consideration for bioinks destined for micro-extrusion printing is shape fidelity. The gels must maintain their printed shape following extrusion, both in the x-y plane and *z*-axis, without causing structural deformation, a particular concern as print height increases [15]. Therefore, gels are often stabilized via crosslinking. Crosslinking can be performed either before or after printing [16,17,18]. This is often accomplished via either the addition of a chemical crosslinker or the initiation of free-radical crosslinking through the application of light, visible or ultraviolet (UV). These methods must also be tuned to have minimal impact on the cells seeded within the bioinks. It is important the researchers take note of potential toxicity caused by the crosslinking process. UV light, in particular, has been shown to lead to cell damage with prolonged exposure. While a popular curing technique, it must be carefully managed to prevent unwanted side effects.

DLP printers are a relatively recent addition to the bioprinting arsenal, entering the scene in 2015 [19]. These printers consist of a build platform that can move in the *z*-axis, a vat with a translucent bottom filled with liquid ink, and a projector, shown in Figure 1B. The DLP system uses projected light to cure bioinks at the bottom of the vat one plane at a time. Unlike extrusion printers that build constructs upwards from the print bed and require inks to support the weight of layers pushing down on them, DLP printers have the curing surface at the bottom of the print. Thus, the materials used must adhere to the layer above them (or the build plate), withstand disassociation from the bottom of the curing vat, and support the weight of additional layers hanging from them as the print grows in size.

These two systems show the most promise in creating objects with sizable z-dimensions and thus have significant potential for creating tissues of implantable size. There is quite a bit of overlap in the properties these systems require from their inks: They should be non-cytotoxic (during printing and post-processing), possess low viscosity during printing (either in the vat or when being extruded through the needle), and be physically stable once on the build surface. Further, the bioinks should withstand cell culture environments (media bath, high temperature), either directly after printing or following a post-processing step. Finally, the bioink must provide a hospitable environment for continued cell growth and maturation. The cells must adhere, expand, and be afforded appropriate signaling during the culture phase. Finding a bioink that meets these biological requirements remains a challenge for researchers today. 

Identifying the composition of the perfect bioink has been a continued debate, much of which depends on the printing process, the types of cells being used, and the mechanical, physical, and chemical environment the system demands. As such, a divide between synthetic, biologic, and combination bioinks has emerged. Synthetic bioinks are excellent at meeting the physical requirements of printing, maintaining shape post-printing and can be modified for appropriate load-bearing properties [21]. The manufactured synthetic bioinks are highly reproducible with minimal batch-to-batch variations. However, synthetics have relatively few biological binding sites and may be cytotoxic either before crosslinking or during crosslinking. In contrast, biological bioinks are derived from natural sources that are often rich in binding sites and can be derived from the same sources as the target tissues researchers want to replicate, leading to high compatibility between the ink and the cell type used. Here we will briefly review some of the most popular biological bioink components. 

## 3. Biological Bioinks

### 3.1. Agarose

Agarose is a polysaccharide made up of 1,3-linked β-galactose and 1,4-linked 3,6-anhydro-α-l-galactose derived from red algae. The molecule can be dissolved easily in hot water, after which the agarose chains form into side-aligned aggregates as the solution cools. These result in an interlocking network of hydrogen bonds, creating a solid gel of agarose chains [22]. Gels can be tuned by using hydroxyethylated agarose for lower strength and melting temperatures, unmodified agarose, or a combination of the two [22]. Agarose on its own is not as cell-friendly as other biologically derived bioinks, presenting low rates of cellular proliferation, cell adhesion/spreading, and biosynthesis of cell components [23,24]. However, this lack of cellular interaction has made agarose an excellent material for creating molds for the 3D formation of cellular aggregates [24]. 

**Figure 2:**
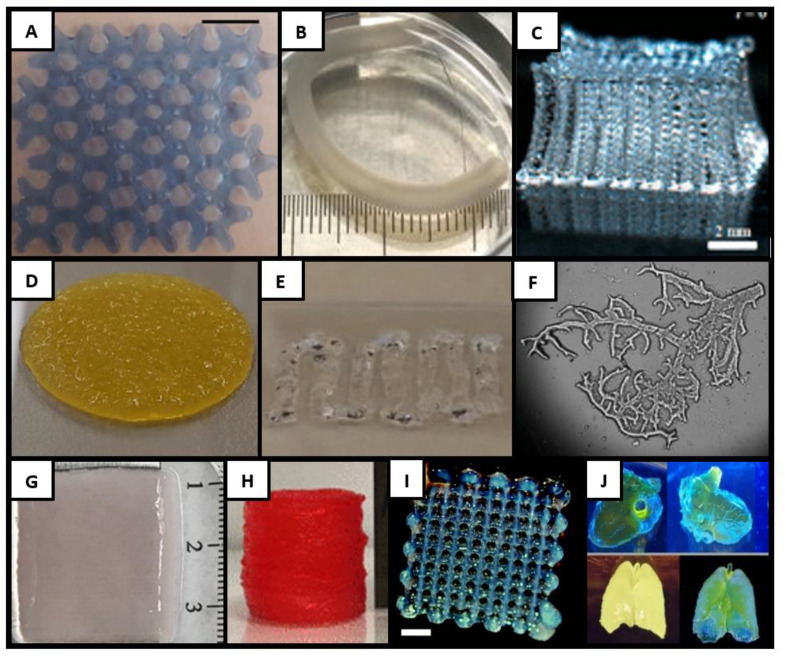
Examples of biologically based bioinks. (**A**) An agarose alginate blend printed for cartilage development [17]. (**B**) Alginate extruded through a triaxial nozzle for blood vessel replacement [32]. (**C**) A large multilayered chitosan construct showing mechanical integrity of printed chitosan processed in an acidic environment [56]. (**D**) Collagen printed over a PCL scaffold (28 mm diameter) for use as a heart valve replacement [65]. (**E**) A combination of ECM and hyaluronic acid gel was seeded with liver spheroids [66]. (**F**) DLP printing of branched ECM [67]. (**G**) A multilayer fibrin skin construct [68]. (**H**) Examples of multilayer gelatin constructs developed for use with osteoblasts [69]. (**I**) Hyaluronic acid extruded with chondrocytes for cartilage engineering [70]. (**J**) Complex structures printed using a DLP printer and silk fibroin ink [71]. Reprinted with the permission from Ref. [17]. Copyright 2018 American Chemical Society; Reprinted with the permission from Ref. [32]. Copyright 2019 AIP Publishing; Reprinted with the permission from Ref. [56]. Copyright 2018 American Chemical Society; Reprinted with the permission from Ref. [65]. Copyright 1969 Elsevier; Reprinted with the permission from Ref. [66]. Copyright 2015 Acta Materialia Inc. Published by Elsevier Ltd; Reprinted with the permission from Ref. [67]. Copyright 2018 Elsevier Ltd; Reprinted with the permission from Ref. [68]. Dr Yoo is an author on this paper and Mary Ann Liebert, Inc. publishers does not require authors of the content being used to obtain a license for their personal reuse of full article, charts/graphs/tables or text excerpt; Reprinted with the permission from Ref. [69]. Copyright 2020 American Chemical Society.; Reprinted with the permission from Ref. [70]. Copyright 2011 American Chemical Society; Reprinted with the permission from Ref. [71]. Copyright 2018 The Author(s).

The physical attributes of agarose have been capitalized on by blending it with other bioink components. It has been combined with alginate, seeded with chondrocytes, and used on an extrusion printer to create honeycomb patterns that maintained cellular viability over 4 weeks, showing potential for cartilage engineering purposes (Figure 2A) [25]. Chemically modified carboxylated agarose (CA) has been used to create bioinks that can be tuned to specific elastic moduli by varying the degree of carboxylation without significantly altering the shear viscosity [26]. CA was shown to increase the survival rate of human mesenchymal stem cells (MSCs) by 33% compared to native agarose. Studies have shown that increasing the stiffness promoted chondrogenesis and maintained cell phenotypes in extrudable gels seeded with human articular chondrocytes [26,27]. In another study, CA was combined with pluronic, a synthetic polymer. Pluronic acted as a sacrificial material to form tubular structures inside a structure with controlled microporosity to develop a system that mimics the architecture of the ECM surrounding a blood vessel [28]. While this group has yet to test structures with cells, CA’s ability to support cellular viability makes this an interesting step forward, as the mechanical properties of agarose can mimic those found in the human body. Agarose has also been used as a support material for freestanding constructs, providing mechanical stability to softer gels such as alginate and gelatin methacrylate (GelMA), which can be cultured while suspended in an agarose slurry to create complex cellularized structures that are supported during cellular maturation but which have easy removal of the support gel post-maturation [29]. While this is not a direct use of agarose as a bioink, it is an important way the molecule can further develop 3D printing processes.

### 3.2. Alginate

Alginate, or alginic acid, is a heteropolysaccharide derived from brown algae [30]. It has a unique ability to form gels through ionic crosslinking when exposed to bipolar ions such as calcium, barium, zinc, and strontium [31,32,33,34]. Consisting of two repeating monomers ((1-4)-β-D-mannuronic acid and α-L-guluronic acid), bioinks developed with this polymer can be tuned for desired mechanical properties by altering the ratio of these monomers [35,36,37,38]. Alginate is available in either a blended form (alginic acid sodium salt, sodium alginate) or as separate monomers (D-mannuronic acid sodium and L-guluronic acid sodium).

Alginate was used for drug and cell delivery long before its first appearance as a bioink, but has since been used for extrusion, LIFT, and inkjet printing applications. The nearly instantaneous ionic crosslinking has made this bioink of particular interest to researchers developing structured tissues, such as tubules, within their prints. Alginate was one of the first bioinks to be used with an extrusion printer fitted with a coaxial nozzle. Extruding alginate through the exterior needle while extruding a crosslinker through the interior allowed researchers to rapidly manufacture microvessel-like tubules throughout their print [39]. Researchers have also used tri-axial nozzles to create multi-layered vessels containing independent layers of human umbilical vein endothelial cells (HUVECs) and human aortic smooth muscle cells, which were implanted into animals as aortic replacements (Figure 2B) [40].

Despite the strengths alginate has as a bioink which can quickly and non-toxically be crosslinked, alginate is a biologically inert molecule with little to no binding moieties for cell interaction and limited pathways for biodegradation. This limits its ability to act as a hospitable growth environment for cells. However, alginate can readily be combined with other biological molecules to create combination gels, improving these characteristics. One study shows that alginate supplemented with carboxymethyl cellulose maintained cellular viability while improving printability and biocompatibility for human MSCs [41]. Nano-fibrillated cellulose has been combined with human chondrocytes and alginate to develop tissue-engineered ears, with the cellulose improving shape fidelity and printing resolution compared to pure alginate [42]. The ionic crosslinking of alginate has proven useful for those pursuing coaxial printing, as alginate blends can be extruded through the outer needle while calcium chloride is extruded through the inner nozzle, creating mechanically hollow tubes, which are being further developed as vascular models and replacements [39,43,44,45,46,47,48,49]. Gelatin alginate hydrogels have been used for skin wound healing by multiple groups, and the combination of structural support from the alginate and improved cellular adhesion from the gelatin has led to cellularized skin constructs that have the potential for enhanced wound healing and accelerated wound closure [50,51,52,53].

### 3.3. Chitosan

Chitosan is a linear polysaccharide molecule derived from the deacetylation of chitin, an acetylated polysaccharide found in fungi, microorganisms, and the shells of crustaceans/insects [54]. The biopolymer was used for many years in tissue engineering as sponge scaffolds, wound dressings, and for cartilage regeneration, among other applications, prior to the advent of bioprinting [55]. It has many unique biological properties that make it enticing for the field, including mucoadhesion; hemostatic activity; the ability to interact with the cell membrane, leading to reorganization of tight junction proteins; antimicrobial properties; analgesic effects; and controllable degradation [55].

Bioprinting with pure chitosan is difficult due to its poor solubility in cell-friendly conditions and low stiffness, reducing its shape fidelity during the printing process. Chitosan precipitates when its solution has a pH above 6.2, making it difficult to keep in solution while maintaining cellular viability (Figure 2C) [56]. However, it can be printed without cells and soaked in a basic solution, either post-printing or between layers, and then seeded with cells after curing [57,58,59]. To allow for a bioink that can directly deposit cells, chitosan can be modified with a carboxymethyl group to improve the solubility at physiological pH. Carboxymethyl-chitosan has been combined with alginate to develop constructs capable of supporting bone MSCs and human induced pluripotent stem cells [60,61]. Chitosan can also be altered through the addition of β-glycerophosphate, which allows the chitosan to remain soluble at a neutral pH and induces thermosensitive gelation at 37 °C. This modified chitosan has been used to print IMR-32 neuroblastoma cells with high post-printing viability [62,63]. Chitosan has also been combined with catechol to create a unique bioink that rapidly solidifies when exposed to serum inks, resulting in a system that can be directly printed into media without the need for external crosslinking mechanisms with high mechanical strength and cell viability [64].

### 3.4. Collagen

When referring to collagen as a bioink, researchers generally refer to collagen type I, a triple helical protein derived from the connective tissue of mammals which has limited variability among species, resulting in minimal immunological reactions [72,73]. Collagen is well known for enhancing cellular attachment and growth, which is attributed to the abundance of integrin-binding domains found on the protein. A unique aspect of this biologic is that collagen remains liquid at low temperatures and gels into a fibrous matrix when exposed to high temperatures (physiological and higher). This gelation is relatively slow—printed collagen can stay liquid for more than 10 min following extrusion and complete gelation can take more than 30 min [72].

These mechanical instabilities make pure collagen a challenging bioink for both micro-extrusion and DLP printers. To overcome this, collagen has been blended with synthetic materials, such as Pluronic, which acts as a support during the printing and gelation phases while collagen improves the cellular environment [74,75]. Synthetic supports such as polycaprolactone (PCL) have been used to enhance the structural integrity of pure collagen—for instance, to bioprint aortic heart valves implanted in mice (Figure 2D) [65]. Collagen has also been blended with other biologics such as GelMA to create a cross-linkable six-layered skin structure that could withstand implantation and showed accelerated wound healing [76]. Blends made with bioceramics or alginates have been used to fabricate bone constructs, and combinations of collagen and heparin have been used to create spinal constructs [77,78,79]. Collagen can also be chemically modified to alter its physical properties while maintaining its biochemical advantages. To this end, methacrylated collagen has been combined with hyaluronic acid to create liver constructs. The combination has been utilized to create a hospitable cell environment that can be stabilized through UV crosslinking for extended in vitro maturation [80]. Recombinant collagen has also been functionalized with a methacrylamide, making it cross-linkable via UV light and thus allowing for use in DLP printers alongside micro-extrusion systems [81].

### 3.5. Extracellular Matrix

Extracellular matrix (ECM) is a complex network containing collagens, elastin, proteoglycans, and glycoproteins extruded by cells, which is highly specific to the tissue where it is produced [82]. Thus, ECM has extreme advantages in creating microenvironments that recapitulate native tissues. The matrix is usually derived through the dissolution of cellular material from tissue (decellularization), using enzymatic, physical, and chemical processes [83]. The decellularized material can be solubilized, resulting in a soft gel that can be bioprinted, and has shown successful outcomes for cellular viability and proliferation/differentiation when printed [84]. However, similarly to other biological components, the gel resulting from pure ECM is soft and has difficulty physically supporting itself. This has led researchers to utilize ECM as a biological component printed into a PCL frame.

Along with PCL frames, ECM has been used as a component of multi-material bioinks that help drive the development of specific tissue types. Bioinks containing HA, gelatin, and crosslinkers (poly-ethylene glycol diacrylate, alkyne, or acrylate) were successfully used to bioprint organoids using ECM sourced tissues such as liver, heart, and skeletal muscle. In one study, the developed liver bioink was successfully used to create extruded liver spheroids that maintained cell functionality during post-printing culture (Figure 2E) [66]. Cardiac ECM was printed in combination with vitamin B2 to allow UVA crosslinking, resulting in mechanically stable constructs that increased cardiomyogenic differentiation when seeded with cardiac progenitor cells [85]. ECM has also been successfully used in DLP printers, combined with GelMA and a photoinitiator, to allow crosslinking when the bioink was exposed to a light source. Researchers used this same base gel with ECM from different tissue sources to create tissue-specific bioinks for the liver and heart, maintaining high viability and improving cellular reorganization post-printing (Figure 2F) [67].

Due to the high efficiency of ECM scaffolds at maintaining and promoting cellular adhesion/growth within their confines, additional work is underway on alternative stabilization techniques. Recent publications have shown that ECM can be stabilized by adding ruthenium and sodium persulfate, compounds that allow the bioink to be crosslinked using visible light following extrusion [86]. These gels were utilized in developing constructs up to 1 cm in size with high cell viability post-printing. ECM can also be methacrylated to create a mono-material ECM bioink which can be stabilized through UV crosslinking post-printing [87]. This material has been used to improve gene expression in skeletal muscle constructs cultured in vitro compared to samples printed using gelatin methacrylate.

### 3.6. Fibrin

Fibrinogen and thrombin, proteins involved in the formation of blood clots, can enzymatically crosslink to result in fibrin. They crosslink into a unique hydrogel with a non-linear elasticity that can be extensively deformed before rupturing [88]. The hydrogel has been used to develop skin grafts, capitalizing on the fact that the proteins are naturally involved in wound healing [89]. It contains amino acid sequences that promote cellular binding, thereby allowing for cell adhesion, growth, and development [90]. Fibrin also has a natural degradation process that promotes the replacement of fibrin with ECM. This is particularly interesting for researchers developing fully degradable constructs that result in new tissue without traces of the implant material. However, the degradation profile of fibrin is rapid due to active cleaving by serine protease plasmin, which limits its use for long-term culturing. In addition, researchers must be cognizant of their fibrin sources, as fibrin from different hosts can result in immune reactions and transmission of infectious diseases [72].

Fibrin poses challenges as a bioink, as pre-crosslinked fibrinogen is very soft with poor shape fidelity, and post-crosslinked fibrin is extremely viscous, making it difficult to extrude [90]. One of the most common techniques to address this is to use pre-crosslinked fibrinogen in multi-material bioinks and crosslink it with thrombin post-printing. Methacrylated and thiolated hyaluronic acid (HA) have been used to increase the stiffness of fibrin gels [91,92]. Gelatin-fibrinogen gels have been used to make liver constructs that were both viable and functional. These gels have been shown to support the coculturing of cardiomyocytes and cardiac fibroblasts [93,94]. Alginate–fibrin blends have been used to create microchips with endothelialized vessels and hepatocytes and in vitro cervical tumor models. This combination takes advantage of the rapid crosslinking of the alginate and the improved biological interactions of fibrin [95,96]. The use of alginate was also shown to elongate the degradation profile of fibrin. The degradation of fibrin can also be delayed through the addition of aprotinin [97,98]. Aprotinin treated fibrinogen gels have been used for wound healing models in vivo implanted for 3 weeks (Figure 2G), as scaffolds for the culture and alignment of Schwann cells, and for the stabilization of human ear-shaped constructs seeded with auricular chondrocytes, which were implanted in vivo for two months [68,99,100].

### 3.7. Gelatin

Gelatin is a denatured collagen protein obtained from animals’ skin, bones, and connective tissues. Sources include pigs, cows, and fish. This protein presents as random coils that can self-associate at low temperatures, thus developing helical structures that lead to a thickening of the material, but this reverts to their randomized conformation when heat is applied [101]. Due to the retention of the Arg–Gly–Asp (RGD) sequence found in non-denatured collagen, gelatin promotes cellular adhesion, proliferation, differentiation, and migration [102]. The ease of transformation due to thermal gelation and excellent cellular environment make this a popular addition to bioinks. Gelatin can thicken a gel during the printing process for extrusion printers allowing for better shape fidelity during printing. In addition, uncrosslinked gelatin can easily be leached out of the system post-printing when combined with other cross-linkable components. This makes gelatin particularly intriguing to groups trying to tune the porosity of their gels post-printing. It also has made gelatin an ideal support material for bioprinting. Gelatin bioinks can be printed into areas intended to be hollow while softer gels are printed around them. Following stabilization of the exterior gel during post-processing, the gelatin is warmed and leached out, leaving hollow space behind. This technique has been of particular interest to those working on the development of vascular networks. Gelatin acts as a support material for many new printing techniques, including freeform reversible embedding of suspended hydrogels and coaxial printing [103,104]. However, the gelatin used as a primary bioink requires additional crosslinking for cellularized prints that need to be maintained post-printing. Many methods of chemically crosslinking gelatin have been explored, but these agents are often cytotoxic, making them unsuitable for bioprinting [72]. Fortunately, several enzymatic crosslinkers, including transglutaminase, have been used to print unmodified gelatin bioinks seeded with HUVECs and human embryonic kidney cells without impacting cellular viability on an extrusion printer [105].

To preserve the positive qualities of gelatin while improving its mechanical characteristics as a bioink, researchers have modified the protein to include a methacrylate group. Gelatin methacrylate (GelMA) preserves many of the biologically-friendly aspects of gelatin, but the addition of the methacrylate group allows for crosslinking through free-radical polymerization, activated by the application of UV light. This can improve structural stability after printing with an extrusion printer and makes GelMA a contender for DLP printing. GelMA maintains high cellular viability with high shape integrity post-printing making it an excellent bioink for larger tissue constructs (Figure 2H) [69]. It has been extensively used in the bioprinting community to create a multitude of constructs, including skin, cartilage, tumors, cornea, blood vessels, and liver and cardiac tissues, capitalizing on the strong bioink–cell interactions and resulting in a bioink that can be used for a wide number of tissues [43,76,106,107,108,109,110,111,112,113,114,115].

### 3.8. Hyaluronic Acid

Hyaluronic acid (HA) is a non-sulfated glycosaminoglycan found in nearly all connective tissues and cartilage [116]. This linear molecule consists of repeating disaccharide units of d-glucuronic acid and N-acetyl-d-glucosamine moieties linked with alternating β-1,3 and β-1,4 glycosidic segments [117]. The three major functional groups primarily control the chemical activity of HA: glucuronic carboxylic acid, an N-acetyl group, and a secondary hydroxyl group [72]. These groups play a major role in HA’s ability to form flexible hydrogels and make it highly biocompatible, particularly for applications that focus on joints such as osteoarthritis [118,119,120,121]. As a molecule, HA has been shown to play a role in early embryonic development, is cell-friendly, and biocompatible throughout its degradation process. As a material for tissue engineering, HA has proven to be highly reproducible, both in its formation and degradation, with mechanics, architecture, and degradation that can easily be manipulated by researchers [72,122]. However, despite the high degree of biocompatibility, HA has relatively poor mechanical properties for bioprinting, a slow gelation rate, and a rapid degradation profile limiting its uses in structures designed for extended periods of culture/use.

Therefore, like many of the bioinks discussed in this article, HA is often used either as a component of a multi-material bioink or is chemically modified before bioprinting. This allows researchers to maintain many of the biological characteristics provided by HA while improving upon the physicochemical properties for a specific use. HA has been combined with synthetic molecules such as hydroxyethyl methacrylate to create UV cross-linkable samples that could easily be printed using an extrusion printer, resulting in strong hydrogels that mimic the viscoelastic properties of natural tissues. These supported chondrocytes post-printing (Figure 2I) [70]. HA can be thiolated and combined with polyethylene glycol (PEG) or methacrylated and combined with GelMA to create photo-cross-linkable bioinks, both of which have been used to fabricated tubular structures reminiscent of vascular structures that supported cells in vitro and in vivo [123,124]. Thiolated HA has also been combined with poly(glycidol)s and extruded alongside alternating strands of PCL to create a strong grid supporting the hydrogel until polymerization. This configuration could extrude both human and equine MSCs, which maintained their viability post-curing for a reported 21 days [125]. HA has also been used in DLP systems, combined with polyurethane to create a structure with mechanical properties similar to articular cartilage. This bioink supported the growth and differentiation of Wharton’s jelly MSCs that were seeded post-printing [126]. A combination of HA and gelatin, both modified with phenolic hydroxyl groups, was also used to print human adipose SCs on an extrusion printer. The construct was crosslinked using visible light and showed tunable stiffness and high cellular viability [127].

### 3.9. Scaffold-Free

Scaffold-free bioinks refer to those systems which use cells solely. In comparison, the other inks presented here act as carriers, seeded with cells as a component of the ink prior to printing. Cell-only inks introduce cells in an already close-packed array during extrusion, allowing cells to quickly self-assemble into aggregates within the printed form [128]. Briefly, this technique utilizing coaxial printing or molding to isolate in long fibers while coalescence occurs. Following the aggregation of cells within the fibers, these strands are then de-molded and re-extruded into the desired 3D shape. The final print is then allowed to fuse and mature [128]. This will enable researchers to capitalize on the ability of cells to self-assemble. In addition, the cell-only nature allows for cell concentrations closer to that seen in vivo compared to bioinks that suspend cells in a carrier. However, this also stipulates that large numbers of cells are needed for this printing methodology, which may inhibit the development of large structures. Further, scaffold-free printing cannot be translated to DLP printing at this time due to the lack of cross-linking ability and remains a solely micro-extrudable option.

The use of scaffold-free bioinks has shown high rates of strand fusion and self-assembly to rapidly create aggregated structures [129]. The technique has been used to create strands from multi-cell cultures and develop co-cultures of βTC-3 cells and fibroblasts, which showed cells self-directing into distinct populations post-printing, maintaining their ability to produce insulin and presenting as a pancreatic tissue model [129]. Porosity has also been introduced into scaffold-free printing through the inclusion of porogens during filament maturation. These porous strands showed increased cell viability and proliferation rates compared to solid cell strands while maintaining the capability to fuse into a single tissue. In addition, porous scaffold-free samples seeded with adipocyte-derived stem cells showed high viability and functionality in scaffold-free printing when differentiated into chondrogenic and osteogenic lineages [130]

### 3.10. Silk

Silk is a unique material for bioprinting in that it consists of two types of proteins—fibroin and sericin, which act as a core and glue within the fiber [131]. Silk fibers are common in the medical field, most familiar as sutures; however, these two proteins can be separated, and both have unique properties that make them attractive for bioink development. Fibroin is a biocompatible hydrophobic protein with a tailorable rate of biodegradation, high mechanical modulus, and the ability to self-assemble into a hydrogel when dissolved in an aqueous solution [132]. It forms thermodynamically stable β-sheets which improve mechanical integrity and slow degradation following gelation [133]. While this gelation process is naturally slow, fibroin can be crosslinked through several cytocompatible methods, enhancing its ability to maintain shape fidelity during the printing process [134]. Sericin is the hydrophilic portion of the silk fiber, often referred to as glue or gum. It is immunologically inert, non-cytotoxic when cultured with cells in vitro, and has been shown to stimulate cell migration/proliferation and collagen production at wound sites facilitating wound healing [135,136,137,138,139]. It can form a hydrogel at low concentrations but has low mechanical strength, and its fragility has limited the use of pure sericin hydrogels as a tissue engineering building block [140,141].

Silk proteins have been incorporated into composite bioinks, modulating their viscosity and gelation to improve printing, allowing researchers to take advantage of their mechanical and biological properties post-printing. The addition of PEG to silk fibroin allowed micro-extrusion constructs to maintain their shapes for 12 weeks in vitro and 6 weeks in vivo when seeded with human bone marrow MSCs [142]. PEG has also been used to crosslink fibroin printed into a support medium, allowing samples to fully stabilize post-printing prior to cell seeding. These constructs supported the growth and differentiation of myoblasts [134]. Combining fibroin with gelatin has been shown to create mechanically stable bioinks that supported chondrogenic development, capitalizing on the entanglement and physical crosslinking of the two gels to stabilize the bioinks without additional crosslinkers [143]. Cartilage-like structures have also been developed, crosslinking fibroin with horseradish peroxidase, used to coculture osteogenic and chondrogenic cells as a strategy for osteochondral defect regeneration [144]. Fibroin has also been shown to be suitable for DLP printing as it can be methacrylated, allowing for light-sensitive cross-linking. Methacrylated silk fibroin was used to prepare complex structures via DLP printing that was mechanically robust and showed high cellular viability and proliferation post-printing (Figure 2J) [71]. Sericin has been combined with GelMA to create highly transparent hydrogels, which were used to cover wounds and allow for real-time monitoring of wound healing [145]. Combinations of fibroin, sericin, and collagen have capitalized on the interaction between the hydrophobic fibroin and hydrophilic sericin to reduce phase separation between the silk and collagen, creating a bioink that was structurally stable for use as a cardiac patch and maintained MSCs during in vitro culture [146].

## 4. Bioinks Today and Tomorrow

Biologics are an important facet of bioink development. As highlighted in this article, their strengths lie in their ability to facilitate cell–cell and cell–construct interactions, maintaining high cell viability, and even providing chemical cues crucial for cellular development through their physical and chemical moieties, as summarized in Table 1. However, it can be easily seen that pure biologics have not proven ideal for 3D printing, primarily due to their physical integrity. As such, continued modification of the components to provide additional mechanical stability is important. Initial work creating methacrylated gelatin and ECM is an exciting first step toward creating strong hydrogels with high biocompatibility. Continued research into alternative crosslinking mechanisms that limit the adverse effects is also an area of current development that will allow further use of bioprinting. Development in benign curing techniques may permit researchers to cure gels further to improve mechanical stability and open the door for bioprinting easily damaged cell lines.

As bioprinting expands, it is not enough to simply develop new bioink options. Those individuals driving bioink development will need in-depth understanding of what their specific systems require—the biologics needed to drive cellular proliferation/maturation and the physical requirements for printing and culturing their construct. This article has highlighted how specific biological components can be chosen and manipulated to provide appropriate, tissue-specific environments. This tailoring should become more finely tuned in the coming years, with researchers worldwide sharing findings on how each component and its blends allow for improved engineering of tissues in the lab.

While the components described in this review are only a few of the many options currently available for bioprinters, we have presented even fewer of the possible alterations and combinations that can be used or formulated to personalize a bioink. This library of biologics, appropriate to bioprinting, will only continue to be expanded and refined. The depths of bioink possibilities are far from plumbed, and the choice of which biologics to include and how to alter/combine them will remain a defining point in bioprinting research as the field evolves.

## Figures and Tables

**Table 1:** Summary of bioinks presented here, with advantages and disadvantages for each bioink. All components are considered in their non-modified (natural) states.

Bioink	Advantages	Disadvantages
*Agarose*	Tunable strength	Low rates of cellular proliferation
	Tunable melting temperatures	Low cell adhesion/spreading
*Alginate*	Tunable strength through alteration of monomer percentages	Biologically inert
	Rapid ionic crosslinking	Limited biodegradability
*Chitosan*	Mucoadhesion	Poor solubility
	Hemostatic activity	Poor shape fidelity post printing
	Interactions with cell membrane	
	Antimicrobial/analgesic effects	
	Controllable degradation	
*Collagen*	Enhanced cellular attachment/growth	Gelation at higher temperatures, liquid form at lower temperatures
*Extracellular Matrix*	Tissue specific	May be difficult to source
	Multitude of growth factors/cell adhesion points	Difficult to characterize
		Batch to batch variability
		Mechanically unstable
*Fibrin*	Enzymatic crosslinking	Rapid degradation profile
	Non-linear elasticity: high deformation potential	Host source may result in immune reaction
	High cell adhesion/growth/development	Poor shape fidelity pre-crosslinking
	Natural degradation	Highly viscous post crosslinking
*Gelatin*	Thermo-reversible gelation	Many crosslinking options are cytotoxic
	High cell adhesion/growth/development	
	Can act as thickening agent/support material for other bioinks	
*Hyaluronic Acid*	High biocompatibility	Poor mechanical properties
	Reproducible/tunable formation and degradation profiles	Slow gelation rate
		Rapid degradation profile
*Scaffold-Free*	High cell density	Complicated manufacturing techniques
	Rapid strand fusion	High cell density (sourcing/expanding)
	Self-assembly	Cannot be used with DLP systems
*Silk (Fibroin)*	Biocompatible	Hydrophobic
	Adjustable degradation	Slow gelation rate
	Mechanically stable	
	Self-assembly	
*Silk (Sericin)*	Immunologically inert	Poor mechanical properties
	Stimulated cell migration/proliferation	
	Gelation at low concentrations	

## References

[1] Thevenot P., Nair A., Dey J., Yang J., Tang L. (2008). Method to analyze three-dimensional cell distribution and infiltration in degradable scaffolds. Tissue Eng. Part C Methods.

[2] Silva M.M., Cyster L.A., Barry J.J., Yang X.B., Oreffo R.O., Grant D.M., Scotchford C.A., Howdle S.M., Shakesheff K.M., Rose F.R. (2006). The effect of anisotropic architecture on cell and tissue infiltration into tissue engineering scaffolds. Biomaterials.

[3] Eltom A., Zhong G. (2019). Muhammad, Scaffold Techniques and Designs in Tissue Engineering Functions and Purposes: A Review. Adv. Mater. Sci. Eng..

[4] Solchaga L.A., Tognana E., Penick K., Baskaran H., Goldberg V.M., Caplan A.I., Welter J.F. (2006). A rapid seeding technique for the assembly of large cell/scaffold composite constructs. Tissue Eng..

[5] Khalil S., Nam J., Sun W. (2005). Multi-nozzle deposition for construction of 3D biopolymer tissue scaffolds. Rapid Prototyp. J..

[6] Thayer P., Martinez H., Gatenholm E., Crooki J.M. (2020). History and Trends of 3D Bioprinting. 3D Bioprinting: Principles and Protocols.

[7] Pardo L., Wilson W.C., Boland T. (2003). Characterization of Patterned Self-Assembled Monolayers and Protein Arrays Generated by the Ink-Jet Method. Langmuir.

[8] Roth E.A., Xu T., Das M., Gregory C., Hickman J.J., Boland T. (2004). Inkjet printing for high-throughput cell patterning. Biomaterials.

[9] Lee V.K., Dias A., Ozturk M.S., Chen K., Tricomi B., Corr D.T., Intes X., Dai G., Turksen K. (2015). 3D Bioprinting and 3D Imaging for Stem Cell Engineering. Bioprinting in Regenerative Medicine.

[10] Sears N.A., Seshadri D.R., Dhavalikar P.S., Cosgriff-Hernandez E. (2016). A Review of Three-Dimensional Printing in Tissue Engineering. Tissue Eng. Part B Rev..

[11] Billiet T., Gevaert E., de Schryver T., Cornelissen M., Dubruel P. (2014). The 3D printing of gelatin methacrylamide cell-laden tissue-engineered constructs with high cell viability. Biomaterials.

[12] Li M., Tian X., Zhu N., Schreyer D.J., Chen X. (2010). Modeling process-induced cell damage in the biodispensing process. Tissue Eng. Part C Methods.

[13] Tirella A., Ahluwalia A. (2012). The impact of fabrication parameters and substrate stiffness in direct writing of living constructs. Biotechnol. Prog..

[14] Buyukhatipoglu K., Jo W., Sun W., Morss C.A. (2009). The role of printing parameters and scaffold biopolymer properties in the efficacy of a new hybrid nano-bioprinting system. Biofabrication.

[15] Paxton N.C., Smolan W., Böck T., Ferry P.W.M., Groll J., Juengst T. (2017). Proposal to assess printability of bioinks for extrusion-based bioprinting and evaluation of rheological properties governing bioprintability. Biofabrication.

[16] Hazur J., Detsch R., Karakaya E., Kaschta J., Teßmar J., Schneidereit D., Friedrich O., Schubert D.W., Boccaccini A.R. (2020). Improving alginate printability for biofabrication: Establishment of a universal and homogeneous pre-crosslinking technique. Biofabrication.

[17] Ouyang L., Highley C.B., Sun W., Burdick J.A. (2017). A Generalizable Strategy for the 3D Bioprinting of Hydrogels from Nonviscous Photo-crosslinkable Inks. Adv. Mater..

[18] Kim E., Seok J.M., Bae S.B., Park S.A., Park W.H. (2021). Silk Fibroin Enhances Cytocompatibilty and Dimensional Stability of Alginate Hydrogels for Light-Based Three-Dimensional Bioprinting. Biomacromolecules.

[19] Shanjani Y., Pan C.C., Elomaa L., Yang Y. (2015). A novel bioprinting method and system for forming hybrid tissue engineering constructs. Biofabrication.

[20] Hu J.B., Tomov M.L., Buikema J.W., Chen C., Mahmoudi M., Wu S.M., Serpooshan V. (2018). Cardiovascular tissue bioprinting: Physical and chemical processes. Appl. Phys. Rev..

[21] Abelardo E., Thomas D.J., Jessop Z.M., Whitaker I.S. (2018). 7-Synthetic material bioinks. 3D Bioprinting for Reconstructive Surgery.

[22] Mayer H.K., Fiechter G., de la Guardia M., Gonzálvez A. (2013). Chapter 10—Electrophoretic Techniques. Comprehensive Analytical Chemistry.

[23] Fedorovich N.E., de Wijn J.R., Verbout A.J., Alblas J., Dhert W.J. (2008). Three-dimensional fiber deposition of cell-laden, viable, patterned constructs for bone tissue printing. Tissue Eng. Part A.

[24] Livoti C.M., Morgan J.R. (2010). Self-assembly and tissue fusion of toroid-shaped minimal building units. Tissue Eng. Part A.

[25] López-Marcial G.R., Zeng A.Y., Osuna C., Dennis J., García J.M., O’Connell G.D. (2018). Agarose-Based Hydrogels as Suitable Bioprinting Materials for Tissue Engineering. ACS Biomater. Sci. Eng..

[26] Forget A., Blaeser A., Miessmer F., Köpf M., Campos D.F.D., Voelcker N.H., Blencowe A., Fischer H., Shastri V.P. (2017). Mechanically Tunable Bioink for 3D Bioprinting of Human Cells. Adv. Healthc. Mater..

[27] Arya N., Forget A., Sarem M., Shastri V.P. (2019). RGDSP functionalized carboxylated agarose as extrudable carriers for chondrocyte delivery. Mater. Sci. Eng. C.

[28] Forget A., Derme T., Mitterberger D., Heiny M., Sweeney C., Mudili L., Dargaville T.R., Shastri V.P. (2019). Architecture-inspired paradigm for 3D bioprinting of vessel-like structures using extrudable carboxylated agarose hydrogels. Emergent Mater..

[29] Mirdamadi E., Muselimyan N., Koti P., Asfour H., Sarvazyan N. (2019). Agarose Slurry as a Support Medium for Bioprinting and Culturing Freestanding Cell-Laden Hydrogel Constructs. 3D Print. Addit. Manuf..

[30] Tariverdian T., Navaei T., Milan P.B., Samadikuchaksaraei A., Mozafari M., Mozafari M., Chauhan N.P.S. (2019). Chapter 16—Functionalized polymers for tissue engineering and regenerative medicines. Advanced Functional Polymers for Biomedical Applications.

[31] Hariyadi D.M., Lin S.C., Wang Y., Bostrom T., Turner M.S., Bhandari B., Coombes A.G. (2010). Diffusion loading and drug delivery characteristics of alginate gel microparticles produced by a novel impinging aerosols method. J. Drug Target.

[32] Mirtič J., Ilaš J., Kristl J. (2018). Influence of different classes of crosslinkers on alginate polyelectrolyte nanoparticle formation, thermodynamics and characteristics. Carbohydr. Polym..

[33] Mørch Ý.A., Donati I., Strand B.L., Skjåk-Bræk G. (2006). Effect of Ca^2+^, Ba^2+^, and Sr^2+^ on Alginate Microbeads. Biomacromolecules.

[34] Straccia M.C., d’Ayala G.G., Romano I., Laurienzo P. (2015). Novel zinc alginate hydrogels prepared by internal setting method with intrinsic antibacterial activity. Carbohydr. Polym..

[35] Gopinathan J., Noh I. (2018). Recent trends in bioinks for 3D printing. Biomater. Res..

[36] Moradali M., Ghods S., Rehm B. (2018). Alginate Biosynthesis and Biotechnological Production.

[37] Draget K.I., Bræk G.S., Smidsrød O. (1994). Alginic acid gels: The effect of alginate chemical composition and molecular weight. Carbohydr. Polym..

[38] Aarstad O., Heggset E.B., Pedersen I.S., Bjørnøy S.H., Syverud K., Strand B.L. (2017). Mechanical Properties of Composite Hydrogels of Alginate and Cellulose Nanofibrils. Polymers.

[39] Gao Q., He Y., Fu J.Z., Liu A., Ma L. (2015). Coaxial nozzle-assisted 3D bioprinting with built-in microchannels for nutrients delivery. Biomaterials.

[40] Gao G., Kim H., Kim B., Kong J., Lee J.Y., Park B.W., Chae S., Kim J., Ban K., Jang J. (2019). Tissue-engineering of vascular grafts containing endothelium and smooth-muscle using triple-coaxial cell printing. Appl. Phys. Rev..

[41] Ahlfeld T., Cidonio G., Kilian D., Duin S., Akkineni A.R., Dawson J.I., Yang S., Lode A., Oreffo R.O.C., Gelinsky M. (2017). Development of a clay based bioink for 3D cell printing for skeletal application. Biofabrication.

[42] Markstedt K., Mantas A., Tournier I., Ávila H.M., Hägg D., Gatenholm P. (2015). 3D Bioprinting Human Chondrocytes with Nanocellulose-Alginate Bioink for Cartilage Tissue Engineering Applications. Biomacromolecules.

[43] Jia W., Gungor-Ozkerim P.S., Zhang Y.S., Yue K., Zhu K., Liu W., Pi Q., Byambaa B., Dokmeci M.R., Shin S.R. (2016). Direct 3D bioprinting of perfusable vascular constructs using a blend bioink. Biomaterials.

[44] Yu Y., Zhang Y., Martin J.A., Ozbolat I.T. (2013). Evaluation of cell viability and functionality in vessel-like bioprintable cell-laden tubular channels. J. Biomech. Eng..

[45] Zhang Y., Yu Y., Chen H., Ozbolat I.T. (2013). Characterization of printable cellular micro-fluidic channels for tissue engineering. Biofabrication.

[46] Attalla R., Ling C., Selvaganapathy P. (2016). Fabrication and characterization of gels with integrated channels using 3D printing with microfluidic nozzle for tissue engineering applications. Biomed. Microdevices.

[47] Zhang Y., Yu Y., Ozbolat I.T. (2013). Direct Bioprinting of Vessel-Like Tubular Microfluidic Channels. J. Nanotechnol. Eng. Med..

[48] Zhang Y., Yu Y., Akkouch A., Dababneh A., Dolati F., Ozbolat I.T. (2015). In Vitro Study of Directly Bioprinted Perfusable Vasculature Conduits. Biomater. Sci..

[49] Gao G., Lee J.H., Jang J., Lee D.H., Kong J.S., Kim B.S., Choi Y.J., Jang W.B., Hong Y.J., Kwon S.M. (2017). Tissue Engineered Bio-Blood-Vessels Constructed Using a Tissue-Specific Bioink and 3D Coaxial Cell Printing Technique: A Novel Therapy for Ischemic Disease. Adv. Funct. Mater..

[50] Liu J., Chi J., Wang K., Liu X., Gu F. (2016). Full-Thickness Wound Healing Using 3D Bioprinted Gelatin-Alginate Scaffolds in Mice: A Histopathological Study.

[51] Shi L., Xiong L., Hu Y., Li W., Chen Z., Liu K., Zhang X. (2018). Three-dimensional printing alginate/gelatin scaffolds as dermal substitutes for skin tissue engineering. Polym. Eng. Sci..

[52] Liu P., Shen H., Zhi Y., Si J., Shi J., Guo L., Shen S.G. (2019). 3D bioprinting and in vitro study of bilayered membranous construct with human cells-laden alginate/gelatin composite hydrogels. Colloids Surf. B Biointerfaces.

[53] Dutta S.D., Hexiu J., Patel D.K., Ganguly K., Lim K.T. (2021). 3D-printed bioactive and biodegradable hydrogel scaffolds of alginate/gelatin/cellulose nanocrystals for tissue engineering. Int. J. Biol. Macromol..

[54] Verma D., Fortunati E., Verma D., Fortunati E., Jain S., Zhang X. (2019). 1-Biopolymer processing and its composites: An introduction. Biomass, Biopolymer-Based Materials, and Bioenergy.

[55] Croisier F., Jérôme C. (2013). Chitosan-based biomaterials for tissue engineering. Eur. Polym. J..

[56] Boza A.M., Wlodarczyk-Biegun M., Campo A., Vázquez-Lasal B., Roman J.S. (2019). Chitosan-based inks: 3D printing and bioprinting strategies to improve shape fidelity, mechanical properties, and biocompatibility of 3D scaffolds. Biomecánica.

[57] Wu Q., Therriault D., Heuzey M.-C. (2018). Processing and Properties of Chitosan Inks for 3D Printing of Hydrogel Microstructures. ACS Biomater. Sci. Eng..

[58] Zhang J., Allardyce B.J., Rajkhowa R., Zhao Y., Dilley R.J., Redmond S.L., Wang X., Liu X. (2018). 3D Printing of Silk Particle-Reinforced Chitosan Hydrogel Structures and Their Properties. ACS Biomater. Sci. Eng..

[59] Almeida C.R., Serra T., Oliveira M.I., Planell J.A., Barbosa M.A., Navarro M. (2014). Impact of 3-D printed PLA- and chitosan-based scaffolds on human monocyte/macrophage responses: Unraveling the effect of 3-D structures on inflammation. Acta Biomater..

[60] Huang J., Fu H., Wang Z., Meng Q., Liu S., Wang H., Zheng X., Dai J., Zhang Z. (2016). BMSCs-laden gelatin/sodium alginate/carboxymethyl chitosan hydrogel for 3D bioprinting. RSC Adv..

[61] Gu Q., Tomaskovic-Crook E., Wallace G.G., Crook J.M. (2017). 3D Bioprinting Human Induced Pluripotent Stem Cell Constructs for In Situ Cell Proliferation and Successive Multilineage Differentiation. Adv. Healthc. Mater..

[62] Roehm K.D., Madihally S.V. (2017). Bioprinted chitosan-gelatin thermosensitive hydrogels using an inexpensive 3D printer. Biofabrication.

[63] Demirtaş T.T., Irmak G., Gümüşderelioğlu M. (2017). A bioprintable form of chitosan hydrogel for bone tissue engineering. Biofabrication.

[64] Lee D., Park J.P., Koh M.-Y., Kim P., Lee J., Shin M., Lee H. (2018). Chitosan-catechol: A writable bioink under serum culture media. Biomater. Sci..

[65] Maxson E.L., Young M.D., Noble C., Go J.L., Heidari B., Khorramirouz R., Morse D.W., Lerman A. (2019). In vivo remodeling of a 3D-Bioprinted tissue engineered heart valve scaffold. Bioprinting.

[66] Skardal A., Devarasetty M., Kang H.-W., Mead I., Bishop C., Shupe T., Lee S.J., Jackson J., Yoo J., Soker S. (2015). A hydrogel bioink toolkit for mimicking native tissue biochemical and mechanical properties in bioprinted tissue constructs. Acta Biomater..

[67] Yu C., Ma X., Zhu W., Wang P., Miller K.L., Stupin J., Koroleva-Maharajh A., Hairabedian A., Chen S. (2019). Scanningless and continuous 3D bioprinting of human tissues with decellularized extracellular matrix. Biomaterials.

[68] Jorgensen A.M., Varkey M., Gorkun A., Clouse C., Xu L., Chou Z., Murphy S.V., Molnar J., Lee S.J., Yoo J.J. (2020). Bioprinted Skin Recapitulates Normal Collagen Remodeling in Full-Thickness Wounds. Tissue Eng. Part A.

[69] Rastin H., Ormsby R.T., Atkins G.J., Losic D. (2020). 3D Bioprinting of Methylcellulose/Gelatin-Methacryloyl (MC/GelMA) Bioink with High Shape Integrity. ACS Appl. Bio Mater..

[70] Pescosolido L., Schuurman W., Malda J., Matricardi P., Alhaique F., Coviello T., van Weeren P.R., Dhert W.J.A., Hennink W.E., Vermonden T. (2011). Hyaluronic Acid and Dextran-Based Semi-IPN Hydrogels as Biomaterials for Bioprinting. Biomacromolecules.

[71] Kim S.H., Yeon Y.K., Lee J.M., Chao J.R., Lee Y.J., Seo Y.B., Sultan M.T., Lee O.J., Lee J.S., Yoon S.-I. (2018). Precisely printable and biocompatible silk fibroin bioink for digital light processing 3D printing. Nat. Commun..

[72] Hospodiuk M., Dey M., Sosnoski D., Ozbolat I.T. (2017). The bioink: A comprehensive review on bioprintable materials. Biotechnol. Adv..

[73] Ferreira A.M., Gentile P., Sartori S., Pagliano C., Cabrele C., Chiono V., Ciardelli G. (2012). Biomimetic soluble collagen purified from bones. Biotechnol. J..

[74] Lee J., Kim G. (2018). Three-Dimensional Hierarchical Nanofibrous Collagen Scaffold Fabricated Using Fibrillated Collagen and Pluronic F-127 for Regenerating Bone Tissue. ACS Appl. Mater. Interfaces.

[75] Homenick C.M., de Silveira G., Sheardown H., Adronov A. (2011). Pluronics as crosslinking agents for collagen: Novel amphiphilic hydrogels. Polym. Int..

[76] Shi Y., Xing T.L., Zhang H.B., Yin R.X., Yang S.M., Wei J., Zhang W.J. (2018). Tyrosinase-doped bioink for 3D bioprinting of living skin constructs. Biomed Mater.

[77] Chen C., Zhao M.L., Zhang R.K., Lu G., Zhao C.Y., Fu F., Sun H.T., Zhang S., Tu Y., Li X.H. (2017). Collagen/heparin sulfate scaffolds fabricated by a 3D bioprinter improved mechanical properties and neurological function after spinal cord injury in rats. J. Biomed. Mater. Res. Part A.

[78] Yang X., Lu Z., Wu H., Li W., Zheng L., Zhao J. (2018). Collagen-alginate as bioink for three-dimensional (3D) cell printing based cartilage tissue engineering. Mater. Sci. Eng. C.

[79] Kim W.J., Yun H.-S., Kim G.H. (2017). An innovative cell-laden α-TCP/collagen scaffold fabricated using a two-step printing process for potential application in regenerating hard tissues. Sci. Rep..

[80] Mazzocchi A., Devarasetty M., Huntwork R., Soker S., Skardal A. (2018). Optimization of collagen type I-hyaluronan hybrid bioink for 3D bioprinted liver microenvironments. Biofabrication.

[81] Tytgat L., Dobos A., Markovic M., van Damme L., van Hoorick J., Bray F., Thienpont H., Ottevaere H., Dubruel P., Ovsianikov A. (2020). High-Resolution 3D Bioprinting of Photo-Cross-linkable Recombinant Collagen to Serve Tissue Engineering Applications. Biomacromolecules.

[82] Yue B. (2014). Biology of the extracellular matrix: An overview. J Glaucoma.

[83] Ott H.C., Matthiesen T.S., Goh S.-K., Black L.D., Kren S.M., Netoff T.I., Taylor D.A. (2008). Perfusion-decellularized matrix: Using nature’s platform to engineer a bioartificial heart. Nat. Med..

[84] Pati F., Jang J., Ha D.-H., Kim S.W., Rhie J.-W., Shim J.-H., Kim D.-H., Cho D.-W. (2014). Printing three-dimensional tissue analogues with decellularized extracellular matrix bioink. Nat. Commun..

[85] Jang J., Kim T.G., Kim B.S., Kim S.-W., Kwon S.-M., Cho D.-W. (2016). Tailoring mechanical properties of decellularized extracellular matrix bioink by vitamin B2-induced photo-crosslinking. Acta Biomater..

[86] Kim H., Kang B., Cui X., Lee S.-H., Lee K., Cho D.-W., Hwang W., Woodfield T.B.F., Lim K.S., Jang J. (2021). Light-Activated Decellularized Extracellular Matrix-Based Bioinks for Volumetric Tissue Analogs at the Centimeter Scale. Adv. Funct. Mater..

[87] Kim W., Lee H., Lee J., Atala A., Yoo J.J., Lee S.J., Kim G.H. (2020). Efficient myotube formation in 3D bioprinted tissue construct by biochemical and topographical cues. Biomaterials.

[88] Janmey P.A., Winer J.P., Weisel J.W. (2009). Fibrin gels and their clinical and bioengineering applications. J. R. Soc. Interface.

[89] Skardal A., Mack D., Kapetanovic E., Atala A., Jackson J.D., Yoo J., Soker S. (2012). Bioprinted amniotic fluid-derived stem cells accelerate healing of large skin wounds. Stem Cells Transl. Med..

[90] de Melo B.A.G., Jodat Y.A., Cruz E.M., Benincasa J.C., Shin S.R., Porcionatto M.A. (2020). Strategies to use fibrinogen as bioink for 3D bioprinting fibrin-based soft and hard tissues. Acta Biomater..

[91] Arulmoli J., Wright H.J., Phan D.T.T., Sheth U., Que R.A., Botten G.A., Keating M., Botvinick E.L., Pathak M.M., Zarembinski T.I. (2016). Combination scaffolds of salmon fibrin, hyaluronic acid, and laminin for human neural stem cell and vascular tissue engineering. Acta Biomater..

[92] Snyder T.N., Madhavan K., Intrator M., Dregalla R.C., Park D. (2014). A fibrin/hyaluronic acid hydrogel for the delivery of mesenchymal stem cells and potential for articular cartilage repair. J. Biol. Eng..

[93] Wei X., Xiaohong W., Yongnian Y., Wei Z., Zhuo X., Feng L., Rendong W., Renji Z. (2007). Rapid Prototyping Three-Dimensional Cell/Gelatin/Fibrinogen Constructs for Medical Regeneration. J. Bioact. Compat. Polym..

[94] Anil Kumar S., Alonzo M., Allen S.C., Abelseth L., Thakur V., Akimoto J., Ito Y., Willerth S.M., Suggs L., Chattopadhyay M. (2019). A Visible Light-Cross-Linkable, Fibrin–Gelatin-Based Bioprinted Construct with Human Cardiomyocytes and Fibroblasts. ACS Biomater. Sci. Eng..

[95] Xu Y., Wang X. (2015). Fluid and cell behaviors along a 3D printed alginate/gelatin/fibrin channel. Biotechnol. Bioeng..

[96] Zhao Y., Yao R., Ouyang L., Ding H., Zhang T., Zhang K., Cheng S., Sun W. (2014). Three-dimensional printing of Hela cells for cervical tumor model in vitro. Biofabrication.

[97] Smith J.D., Chen A., Ernst L.A., Waggoner A.S., Campbell P.G. (2007). Immobilization of Aprotinin to Fibrinogen as a Novel Method for Controlling Degradation of Fibrin Gels. Bioconjugate Chem..

[98] Lorentz K.M., Kontos S., Frey P., Hubbell J.A. (2011). Engineered aprotinin for improved stability of fibrin biomaterials. Biomaterials.

[99] Ning L., Sun H., Lelong T., Guilloteau R., Zhu N., Schreyer D.J., Chen X. (2018). 3D bioprinting of scaffolds with living Schwann cells for potential nerve tissue engineering applications. Biofabrication.

[100] Kang H.-W., Lee S.J., Ko I.K., Kengla C., Yoo J.J., Atala A. (2016). A 3D bioprinting system to produce human-scale tissue constructs with structural integrity. Nat. Biotechnol..

[101] Chiou B.-S., Avena-Bustillos R.J., Bechtel P.J., Jafri H., Narayan R., Imam S.H., Glenn G.M., Orts W.J. (2008). Cold water fish gelatin films: Effects of cross-linking on thermal, mechanical, barrier, and biodegradation properties. Eur. Polym. J..

[102] Sakai S., Hirose K., Taguchi K., Ogushi Y., Kawakami K. (2009). An injectable, in situ enzymatically gellable, gelatin derivative for drug delivery and tissue engineering. Biomaterials.

[103] Hinton T.J., Jallerat Q., Palchesko R.N., Park J.H., Grodzicki M.S., Shue H.-J., Ramadan M.H., Hudson A.R., Feinberg A.W. (2015). Three-dimensional printing of complex biological structures by freeform reversible embedding of suspended hydrogels. Science Adv..

[104] Lee A., Hudson A.R., Shiwarski D.J., Tashman J.W., Hinton T.J., Yerneni S., Bliley J.M., Campbell P.G., Feinberg A.W. (2019). 3D bioprinting of collagen to rebuild components of the human heart. Science.

[105] Irvine S.A., Agrawal A., Lee B.H., Chua H.Y., Low K.Y., Lau B.C., Machluf M., Venkatraman S. (2015). Printing cell-laden gelatin constructs by free-form fabrication and enzymatic protein crosslinking. Biomed. Microdevices.

[106] Ding H., Illsley N.P., Chang R.C. (2019). 3D Bioprinted GelMA Based Models for the Study of Trophoblast Cell Invasion. Sci. Rep..

[107] Zhou X., Zhu W., Nowicki M., Miao S., Cui H., Holmes B., Glazer R.I., Zhang L.G. (2016). 3D Bioprinting a Cell-Laden Bone Matrix for Breast Cancer Metastasis Study. ACS Appl. Mater. Interfaces.

[108] Kilic Bektas C., Hasirci V. (2019). Cell Loaded 3D Bioprinted GelMA Hydrogels for Corneal Stroma Engineering. Biomater. Sci..

[109] Ruiz-Cantu L., Gleadall A., Faris C., Segal J., Shakesheff K., Yang J. (2020). Multi-material 3D bioprinting of porous constructs for cartilage regeneration. Mater. Sci. Eng. C.

[110] Mouser V.H., Melchels F.P., Visser J., Dhert W.J., Gawlitta D., Malda J. (2016). Yield stress determines bioprintability of hydrogels based on gelatin-methacryloyl and gellan gum for cartilage bioprinting. Biofabrication.

[111] Bejleri D., Streeter B.W., Nachlas A.L.Y., Brown M.E., Gaetani R., Christman K.L., Davis M.E. (2018). A Bioprinted Cardiac Patch Composed of Cardiac-Specific Extracellular Matrix and Progenitor Cells for Heart Repair. Adv. Healthc. Mater..

[112] Koti P., Muselimyan N., Mirdamadi E., Asfour H., Sarvazyan N.A. (2019). Use of GelMA for 3D printing of cardiac myocytes and fibroblasts. J. 3D Print. Med..

[113] Bhise N.S., Manoharan V., Massa S., Tamayol A., Ghaderi M., Miscuglio M., Lang Q., Zhang Y.S., Shin S.R., Calzone G. (2016). A liver-on-a-chip platform with bioprinted hepatic spheroids. Biofabrication.

[114] Ma X., Qu X., Zhu W., Li Y.-S., Yuan S., Zhang H., Liu J., Wang P., Lai C.S.E., Zanella F. (2016). Deterministically patterned biomimetic human iPSC-derived hepatic model via rapid 3D bioprinting. Proc. Natl. Acad. Sci. USA.

[115] Cuvellier M., Ezan F., Oliveira H., Rose S., Fricain J.C., Langouët S., Legagneux V., Baffet G. (2021). 3D culture of HepaRG cells in GelMa and its application to bioprinting of a multicellular hepatic model. Biomaterials.

[116] Zhang L., Hu J., Athanasiou K.A. (2009). The role of tissue engineering in articular cartilage repair and regeneration. Crit. Rev. Biomed. Eng..

[117] Zhong S.P., Campoccia D., Doherty P.J., Williams R.L., Benedetti L., Williams D.F. (1994). Biodegradation of hyaluronic acid derivatives by hyaluronidase. Biomaterials.

[118] Migliore A., Granata M. (2008). Intra-articular use of hyaluronic acid in the treatment of osteoarthritis. Clin. Interv. Aging.

[119] Bowman S., Awad M.E., Hamrick M.W., Hunter M., Fulzele S. (2018). Recent advances in hyaluronic acid based therapy for osteoarthritis. Clin. Transl. Med..

[120] Migliore A., Tormenta S., Massafra U., Bizzi E., Iannessi F., Alimonti A., Granata M. (2008). Intra-articular administration of hylan G-F 20 in patients with symptomatic hip osteoarthritis: Tolerability and effectiveness in a large cohort study in clinical practice. Curr. Med. Res. Opin..

[121] Luo Y., Kirker K.R., Prestwich G.D. (2000). Cross-linked hyaluronic acid hydrogel films: New biomaterials for drug delivery. J. Control. Release.

[122] Khunmanee S., Jeong Y., Park H. (2017). Crosslinking method of hyaluronic-based hydrogel for biomedical applications. J. Tissue Eng..

[123] Skardal A., Zhang J., McCoard L., Xu X., Oottamasathien S., Prestwich G.D. (2010). Photocrosslinkable hyaluronan-gelatin hydrogels for two-step bioprinting. Tissue Eng. Part A.

[124] Skardal A., Zhang J., McCoard L., Oottamasathien S., Prestwich G.D. (2010). Dynamically Crosslinked Gold Nanoparticle–Hyaluronan Hydrogels. Adv. Mater..

[125] Stichler S., Böck T., Paxton N., Bertlein S., Levato R., Schill V., Smolan W., Malda J., Teßmar J., Blunk T. (2017). Double printing of hyaluronic acid/poly(glycidol) hybrid hydrogels with poly(ε-caprolactone) for MSC chondrogenesis. Biofabrication.

[126] Shie M.-Y., Chang W.-C., Wei L.-J., Huang Y.-H., Chen C.-H., Shih C.-T., Chen Y.-W., Shen Y.-F. (2017). 3D Printing of Cytocompatible Water-Based Light-Cured Polyurethane with Hyaluronic Acid for Cartilage Tissue Engineering Applications. Materials.

[127] Sakai S., Ohi H., Hotta T., Kamei H., Taya M. (2018). Differentiation potential of human adipose stem cells bioprinted with hyaluronic acid/gelatin-based bioink through microextrusion and visible light-initiated crosslinking. Biopolymers.

[128] Yu Y., Moncal K.K., Li J., Peng W., Rivero I., Martin J.A., Ozbolat I.T. (2016). Three-dimensional bioprinting using self-assembling scalable scaffold-free “tissue strands” as a new bioink. Sci. Rep..

[129] Akkouch A., Yu Y., Ozbolat I.T. (2015). Microfabrication of scaffold-free tissue strands for three-dimensional tissue engineering. Biofabrication.

[130] Wu Y., Hospodiuk M., Peng W., Gudapati H., Neuberger T., Koduru S., Ravnic D.J., Ozbolat I.T. (2018). Porous tissue strands: Avascular building blocks for scalable tissue fabrication. Biofabrication.

[131] Oliveira J.M. (2020). Current and future trends of silk fibroin-based bioinks in 3D printing. J. 3D Print. Med..

[132] Gupta S., Alrabaiah H., Christophe M., Rahimi-Gorji M., Nadeem S., Bit A. (2021). Evaluation of silk-based bioink during pre and post 3D bioprinting: A review. J. Biomed. Mater. Res. Part B Appl. Biomater..

[133] Matsumoto A., Chen J., Collette A.L., Kim U.-J., Altman G.H., Cebe P., Kaplan D.L. (2006). Mechanisms of Silk Fibroin Sol−Gel Transitions. J. Phys. Chem. B.

[134] Rodriguez M.J., Dixon T.A., Cohen E., Huang W., Omenetto F.G., Kaplan D.L. (2018). 3D freeform printing of silk fibroin. Acta Biomater..

[135] Panilaitis B., Altman G.H., Chen J., Jin H.J., Karageorgiou V., Kaplan D.L. (2003). Macrophage responses to silk. Biomaterials.

[136] Terada S., Nishimura T., Sasaki M., Yamada H., Miki M. (2002). Sericin, a protein derived from silkworms, accelerates the proliferation of several mammalian cell lines including a hybridoma. Cytotechnology.

[137] Terada S., Sasaki M., Yanagihara K., Yamada H. (2005). Preparation of silk protein sericin as mitogenic factor for better mammalian cell culture. J. Biosci. Bioeng..

[138] Aramwit P., Palapinyo S., Srichana T., Chottanapund S., Muangman P. (2013). Silk sericin ameliorates wound healing and its clinical efficacy in burn wounds. Arch. Derm. Res..

[139] Aramwit P., Sangcakul A. (2007). The effects of sericin cream on wound healing in rats. Biosci. Biotechnol. Biochem..

[140] Mandal B.B., Priya A.S., Kundu S.C. (2009). Novel silk sericin/gelatin 3-D scaffolds and 2-D films: Fabrication and characterization for potential tissue engineering applications. Acta Biomater..

[141] Teramoto H., Nakajima K., Takabayashi C. (2005). Preparation of elastic silk sericin hydrogel. Biosci. Biotechnol. Biochem..

[142] Zheng Z., Wu J., Liu M., Wang H., Li C., Rodriguez M.J., Li G., Wang X., Kaplan D.L. (2018). 3D Bioprinting of Self-Standing Silk-Based Bioink. Adv. Healthc. Mater..

[143] Singh Y.P., Bandyopadhyay A., Mandal B.B. (2019). 3D Bioprinting Using Cross-Linker-Free Silk–Gelatin Bioink for Cartilage Tissue Engineering. ACS Appl. Mater. Interfaces.

[144] Ribeiro V.P., Pina S., Costa J.B., Cengiz I.F., García-Fernández L., Fernández-Gutiérrez M.D.M., Paiva O.C., Oliveira A.L., San-Román J., Oliveira J.M. (2019). Enzymatically Cross-Linked Silk Fibroin-Based Hierarchical Scaffolds for Osteochondral Regeneration. ACS Appl. Mater. Interfaces.

[145] Chen C.-S., Zeng F., Xiao X., Wang Z., Li X.-L., Tan R.-W., Liu W.-Q., Zhang Y.-S., She Z.-D., Li S.-J. (2018). Three-Dimensionally Printed Silk-Sericin-Based Hydrogel Scaffold: A Promising Visualized Dressing Material for Real-Time Monitoring of Wounds. ACS Appl. Mater. Interfaces.

[146] Sanz-Fraile H., Amoros S., Mendizabal I., Galvez-Monton C., Prat-Vidal C., Bayes-Genis A., Navajas D., Farre R., Otero J. (2020). Silk-Reinforced Collagen Hydrogels with Raised Multiscale Stiffness for Mesenchymal Cells 3D Culture. Tissue Eng. Part A.

